# Clinical characteristics and prognostic risk factors of mortality in patients with interstitial lung diseases and viral infection: a retrospective cohort study

**DOI:** 10.1099/jmm.0.001449

**Published:** 2021-11-05

**Authors:** Lijuan Li, Chulei Wang, Lingxiao Sun, Xiaoqi Zhang, Guoru Yang

**Affiliations:** ^1^​ Department of Pulmonary and Critical Care Medicine, National Center for Clinical Research on Respiratory Diseases, China–Japan Friendship Hospital, Beijing 100029, PR China; ^2^​ Department of Pulmonary and Critical Care Medicine, Second People’s Hospital of Weifang, Weifang 261041, PR China

**Keywords:** interstitial lung disease, immunocompromised, prognosis, viral infection

## Abstract

**Introduction:**

Patients with interstitial lung disease (ILD) who subsequently develop a viral infection have high rates of morbidity and mortality.

**Hypothesis/Gap statement:**

Few large-scale epidemiological studies have investigated potential prognostic factors for morbidity and mortality in this patient group.

**Aim:**

To evaluate the risk factors for morbidity and mortality in hospitalized patients with ILD and viral infection, as well as the clinical characteristics.

**Methodology:**

This retrospective cohort study included patients with ILD who were hospitalized for a viral infection in two tertiary academic hospitals in China, between 1 January 2013 and 31 December 2019. We analysed the prevalence of comorbidities, clinical characteristics, 30 day mortality rates, and prognostic risk factors.

**Results:**

A total of 282 patients were included; 195 and 87 were immunocompromised and immunocompetent, respectively. The most common underlying interstitial diseases were idiopathic pulmonary fibrosis (42.9 %) and connective tissue disease (36.9 %). The 30 day mortality rate was 20.6 %. During the influenza season, an increase in influenza virus (IFV) (25.7 %), respiratory syncytial virus (14.9 %) and cytomegalovirus (CMV) (11.3 %) cases was observed in the immunocompromised group. The most frequently detected virus in the immunocompetent group was IFV (44.8 %), followed by respiratory syncytial virus (11.5 %), and human rhinovirus (9.2 %). During the non-influenza season, CMV (34.4 %) was the main virus detected in the immunocompromised group. The 30 day mortality rates of non-IFV patients were higher than those of IFV patients. Older age (>60 years), respiratory failure, persistent lymphocytopenia, invasive mechanical ventilation and non-IFV virus infection were significantly associated with increased 30 day mortality.

**Conclusion:**

Patients with ILD who develop viral infection have high rates of morbidity and mortality, which are associated with increased age (>60 years), respiratory failure, mechanical ventilation, persistent lymphocytopenia and non-IFV virus infection. These risk factors should be carefully considered when determining treatment strategies for this patient population.

## Introduction

Few studies have evaluated the impact of viral infections on the acute exacerbation of idiopathic pulmonary fibrosis (IPF) and/or non-IPF interstitial lung disease (ILD). Saraya *et al*. documented respiratory virus infections in 19.2 % of patients with acute exacerbation of interstitial pneumonia; no difference was observed between patients with IPF and non-IPF ILD [1]. In another study in which bronchoalveolar lavage was performed in 18 patients presenting with an acute decline in fibrotic lung disease, five had culture or PCR evidence of viral infection [one parainfluenza virus [PIV] case, two herpes simplex virus cases and two cytomegalovirus (CMV) cases] [2].

The acute exacerbation of IPF is a dangerous condition and has a mortality rate of over 50 % [3]. Some reports have documented 1 year mortality rates of almost 100 % in patients with an acute exacerbation of IPF [4, 5]. Weng found that 60 % of samples collected from patients with an acute exacerbation of IPF were virus positive [6]. Drake *et al*. concluded that patients with ILD, particularly those with poor lung function and obesity, are at an increased risk of death from coronavirus disease [7]. However, there is a current lack of large-scale epidemiological studies that have investigated viral infections and prognosis in patients with ILD. Therefore, the purpose of this study was to evaluate potential risk factors for mortality in hospitalised patients with ILD and viral infections, as well as clinical characteristics.

## Methods

### Study design and participants

We retrospectively recruited patients with an acute exacerbation of ILD and viral infection, who were hospitalized between 1 January 2016 and 31 December 2019, at two secondary and tertiary academic hospitals in China. IPF was defined by the 2007 American Thoracic Society/European Respiratory Society criteria [8]; the definition was broadened to include patients with previously known or established fibrotic disease at admission [9]. We enrolled patients who had usual interstitial pneumonia patterns on their radiological examination, meaning those with an acute exacerbation of connective tissue disease (CTD)-associated interstitial pneumonia and unilateral lung transplantation for ILD. The inclusion criteria were as follows: (1) previous ILD; (2) acute respiratory infection symptoms, including fever, cough, expectoration or dyspnoea; (3) presence of new bilateral pulmonary ground-glass abnormalities, with consolidation superimposed on a background of a reticular and/or honeycomb pattern on chest computed tomography; and (4) positive viral nucleic acid obtained from nasopharyngeal swabs, sputum or bronchoalveolar lavage fluid (BALF). Patients without evidence of viral infection or a prior history of ILD were excluded.

### Study quality control

Key investigators, including clinicians, statisticians, microbiologists and radiologists, worked together to draft the protocol and create a single formatted case report form (CRF) used by all centres. Before study initiation, all investigators from the six centres received training related to the study protocol, including the screening process, definitions of underlying diseases, and the formatted CRF. After the data were collected, CRFs were reviewed by a trained researcher to ensure completeness and data quality. The study was approved by the Ethics Committee of China–Japan Friendship Hospital. There was a centralized collaboration between all participating hospitals, which included anonymized data submission and collection.

### Data collection

The following data were collected from the medical records of patients during their hospitalisation: (1) demographics; (2) clinical symptoms; (3) initial vital signs and lung examination findings; (4) severity of disease (indicated by intensive care unit [ICU] admission, use of invasive or non-invasive mechanical ventilation, pneumonia severity index (PSI) score and/or confusion-urea-respiratory rate-blood pressure-65 (CURB-65) score [10, 11]; (5) laboratory and microbiological data (blood, sputum and/or BALF samples, bacterial or fungal cultures, viral nucleic acid detection and antibiotic susceptibility patterns); (6) treatment information, including use of vasoactive agents, antimicrobials, glucocorticoids and/or other immunosuppressants; and (7) survival status 30 days after admission. High-dose steroid use within 30 days of admission was defined as a prednisolone or glucocorticoid dose of at least 30 mg/day. Persistent lymphocytopenia was defined as a peripheral blood lymphocyte count of <1×10^9^ l^−1^ for more than 7 days.

### Diagnostic procedures

A viral aetiology was confirmed based on the following criteria: reverse transcription real-time (RT)-PCR (Shanghai Zhijiang Biological Technology, China) detection of respiratory viruses, including CMV, respiratory syncytial virus (RSV), influenza virus (IFV) types A and B, PIV, human rhinovirus (HRV), human metapneumovirus, adenovirus and *Pneumocystis jirovecii* in sputum, endotracheal aspirate; from the BALF or nasopharyngeal swabs. Bacteria or atypical pathogens were confirmed if one of the following criteria were met: (1) positive bacterial culture; (2) positive urinary antigen for *Legionella pneumophila* (Binax Now; Trinity Biotech, Bray, Ireland) or *Streptococcus pneumoniae* (Binax Now; Emergo Europe, Amsterdam, The Netherlands); and (3) detection of *Mycoplasma pneumoniae *, *Chlamydia pneumoniae* or *L. pneumophila* in sputum, BALF, endotracheal aspirate or nasopharyngeal swabs using RT-PCR. The Platelia *Aspergillus* test was used for galactomannan detection (Bio-Rad Laboratories, Marnes-la-Coquette, France).

### Pathogen-specific diagnostic information

A diagnosis of pneumonia caused by *Aspergillus* required one or more of the following criteria: (1) histopathologic or direct microscopic evidence of dichotomous septate hyphae with a tissue culture positive for *Aspergillus*; (2) a positive *Aspergillus* culture from BALF; (3) a galactomannan optical index in BALF of ≥1; (4) a galactomannan optical index in serum of ≥0.5; and (5) *Aspergillus* species identified by culture and microscopic characteristics [12, 13].

The diagnosis of *Pneumocystis jirovecii* pneumonia (PCP) was based on one of the following criteria: (1) high-resolution computed tomography imaging showing diffuse ground-glass opacity with a patchy distribution; (2) mycological criteria (microscopic examination of the respiratory sample revealing the presence of *Pneumocystis* cystic or trophic forms); and (3) a positive PCR test for *Pneumocystis* deoxyribonucleic acid [14].

Co-infection was documented if bacteria or fungi were isolated from lower respiratory tract specimens (qualiﬁed sputum, endotracheal aspirate and BALF) and/or blood samples within 48 h of hospitalization. Nosocomial infection was diagnosed based on clinical signs or symptoms of nosocomial pneumonia, bacteremia, and a positive culture of a new pathogen obtained from lower respiratory tract specimens and/or blood samples obtained ≥48 h after admission.

### Statistical analysis

Patient demographics, clinical characteristics and pathogen testing results are expressed as mean (±standard deviation), median (interquartile range) or number (percentage). Group comparisons were conducted using Student’s *t*-test or the Wilcoxon rank-sum test for continuous variables with and without normal distributions, respectively. Categorical variables of the two groups were compared using the *χ*2 test. Cox regression analysis was used to examine independent predictors of mortality, and its results were reported as hazard ratio (HR) and 95 % CI. Kaplan–Meier survival curves were used to compare the 30 day survival rate for patients by the log-rank test.

Statistical analyses were performed using SPSS version 19.0 (SPSS, Chicago, IL, USA). All tests were two-sided, and *P*-values<0.05 were considered statistically significant.

### Patient and public involvement

Neither patients nor the public were involved in the development of the research question, study design, patient recruitment or the conduct of the study.

## Results

A total of 282 patients with ILD who developed viral infection between 1 January 2013 and 31 December 2019 were identified. Approximately 36 % of the patients were women, with a median age of 65.0. The main symptoms included fever (75.4 %), cough (94.8 %), expectoration (70.8 %) and dyspnoea (67.2 %). The most common underlying interstitial-related diseases were IPF (42.9 %), CTD (36.9 %), chronic obstructive pulmonary disease (8.9 %) and ILD requiring unilateral lung transplantation (10.6 %). Ninety-five (43.3 %) patients were admitted to the ICU for treatment, with 23.8% and 24.8 % having received non-invasive and invasive ventilation, respectively. The 30 day mortality rates were 20.6 %, respectively. A total of 195 patients were immunocompromised, and 87 patients were immunocompetent. The following parameters were significantly higher in the immunocompromised group than in the immunocompetent group: proportion of patients with persistent lymphocytopenia, diabetes and CTD; use of anti-* Pseudomonas* drugs, anti-*Aspergillus* drugs, ganciclovir and sulfonamides; requirement for ICU admission, non-invasive ventilation, invasive mechanical ventilation and/or extracorporeal membrane oxygenation; adverse outcomes including respiratory failure and septic shock; peripheral blood leucocyte, neutrophil and lymphocyte counts; and lactate dehydrogenase, urea nitrogen, d-dimer and procalcitonin levels (*P*<0.05). Age, haemoglobin levels and the proportion of patients with cough symptoms and IPF were significantly lower in the immunocompromised group than in the immunocompetent group (Table 1).

**Table 1.** Clinical characteristics of viral pneumonia with interstitial lung disease between immunocompetent and immunocompromised group

Variables	Total, *N*=282	Immunocompromised group, *n*=195	Immunocompetent group, *n*=87	*P*-value
Sex, female, *n* (%)	112 (39.7)	78 (40.0)	27 (31.0)	0.680
Age, median (IQR)	65.0 (56.0–72.0)	62.0 (53.5–69.0)	69.0 (63.0–76.0)	<0.001
Symptoms and signs, *n* (%)				
Fever	181 (64.2)	128 (65.6)	53 (60.9)	0.445
Cough	270 (95.7)	183 (93.8)	87 (100.0)	0.018
Expectoration	256 (90.8)	175 (89.7)	81 (93.1)	0.368
Dyspnoea	218 (77.3)	151 (77.4)	67 (77.0)	0.937
Laboratory examination				
White blood cell,×10^9^ l^−1^ (IQR)	7.81 (5.73–11.04)	8.47 (5.92–9.67)	7.31 (5.38–9.17)	0.007
Neutrophils,×10^9^ l^−1^ (IQR)	6.17 (3.95–8.88)	6.74 (4.57–9.67)	5.09 (3.31–6.90)	<0.001
Lymphocyte,×10^9^ l^−1^ (IQR)	1.10 (0.69–1.64)	1.00 (0.60–1.51)	1.37 (0.94–1.82)	0.001
Persistent lymphocytopenia	101 (35.8)	81 (41.5)	20 (23.0)	0.003
Mean hemoglobin ±sd, g l^−1^	120.5±23.8	116.0±24.6	128.7±20.0	<0.001
Mean albumin ±sd, g l^−1^	35.2±5.3	34.7±5.2	35.9±5.5	0.078
Lactate dehydrogenase, U l^−1^	303 (224–433)	328 (254–472)	254 (200–333)	<0.001
Blood urea nitrogen, mmol l^−1^	5.61 (4.10–8.15)	6.03 (4.30–10.42)	4.80 (3.94–6.23)	<0.001
d-dimer, mmol l^−1^	1.05 (0.40–2.89)	1.10 (0.42–2.22)	0.46 (0.16–1.37)	<0.001
Procalcitonin, ng ml^−1^	0.24 (0.09–0.39)	0.27 (0.11–0.43)	0.14 (0.07–0.30)	0.002
Oxygenation index	274.0 (167.6–358.0)	267.3 (142.1–357.0)	282.0 (212.3–358.0)	0.110
Severe pneumonia index score	75 (63–98)	82 (62–106)	69 (63–84)	0.055
CURB65 score >1	75 (26.6)	55 (28.2)	20 (23.0)	0.360
Underlying diseases, *n* (%)				
Diabetes mellitus	80 (28.4)	64 (32.8)	16 (18.4)	0.013
Connective tissue disease*	104 (36.9)	92 (47.2)	12 (13.8)	<0.001
Idiopathic pulmonary fibrosis	121 (42.9)	63 (32.3)	58 (66.7)	<0.001
Chronic obstructive pulmonary disease	25 (8.9)	12 (6.2)	13 (14.9)	0.016
Radiotherapy and chemotherapy of malignant solid tumour	4 (1.4)	4 (2.1)	0 (0)	0.178
Unilateral lung transplantation†	30 (10.6)	30 (15.4)	0 (0)	<0.001
Current smoker or ex-smoker	109 (38.7)	66 (33.8)	43 (49.4)	0.013
Bronchoalveolar lavage, *n* (%)	157 (55.7)	117 (60.0)	40 (46.0)	0.029
Treatment, before admission, *n* (%)				
Antibiotics	194 (68.8)	132 (67.7)	62 (71.3)	0.550
Antiviral drugs	52 (18.4)	38 (19.5)	14 (16.1)	0.497
Treatment, during hospitalization, *n* (%)				
Anti - Pseudomonas aeruginosa drugs	198 (70.2)	145 (74.4)	53 (60.9)	<0.001
Voriconazole or caspofungin	100 (35.5)	91 (46.7)	9 (10.3)	<0.001
Ganciclovir	120 (42.6)	113 (57.9)	7 (8.0)	<0.001
Trimethoprim	103 (36.5)	101 (51.8)	2 (2.3)	<0.001
Complications, *n* (%)				
Noninvasive ventilation	67 (23.8)	54 (27.7)	13 (14.9)	0.020
Invasive mechanical ventilation	70 (24.8)	57 (29.2)	13 (14.9)	0.010
Mechanical ventilation	99 (35.1)	77 (39.5)	22 (25.3)	0.021
Respiratory failure	137 (48.6)	108 (55.4)	29 (33.3)	0.001
ICU admission	95 (33.7)	80 (41.0)	15 (17.2)	<0.001
Septic shock during hospitalization	47 (16.7)	43 (22.1)	4 (4.6)	<0.001
Extracorporeal membrane oxygenation	19 (6.7)	17 (8.7)	2 (2.3)	0.047
30 day mortality	58 (20.6)	46 (23.6)	12 (13.8)	0.060

*Connective tissue disorders: rheumatoid arthritis, systemic lupus erythematosus, dermatomyositis, polymyositis, systemic sclerosis, Sjogren’s syndrome, etc.

†The reason of unilateral lung transplantation was interstitial lung disease.

‡Other interstitial pneumonia includes non-specific interstitial pneumonia, organizing pneumonia, allergic pneumonia, radiation pneumonia, drug-induced interstitial pneumonia, etc.

During the influenza season (November, December, January, February), an increase in IFV (25.7 %), RSV (14.9 %) and CMV (11.3 %) cases was found in the immunocompromised group. The most frequently detected virus in the immunocompetent group was IFV (44.8 %), followed by RSV (11.5%) and HRV (9.2 %). During the non-influenza season, CMV (34.4 %) was the main virus detected in the immunocompromised group. No dominant virus type was observed in the immunocompetent group; the most frequently detected virus was IFV (15.9 %), followed by adenovirus (5.7 %), HRV (4.6 %), RSV (3.4 %) and PIV (2.3 %) (Table 2). In immunocompromised patients, bacteria (13.8 %), *Pneumocystis jirovecii* (12.8 %) and *Aspergillus* (11.8 %) were the most frequently detected pathogens; the most isolated bacteria were *Staphylococcus aureus* (3.6 %), *Klebsiella pneumoniae* (3.1 %) and *Pseudomonas aeruginosa* (3.1 %). In the immunocompetent group, *Aspergillus* (6.9 %), bacteria (3.4 %) and *Mycoplasma* (2.3 %) were the dominant pathogens. Secondary nosocomial bacterial infections were most frequently attributed to *Acinetobacter baumannii *, *Pseudomonas aeruginosa* and *Klebsiella pneumoniae* (Table 2).

**Table 2.** Pathogen results of pneumonia between the immunocompetent and immunocompromised group

Variables, *n* (%)	Immunocompromised group, *n*=195	Immunocompetent group, *n*=87	*P*- Value
One virus	161 (82.6)	80 (92.0)	0.039
Two or more viruses	34 (17.4)	7 (8.0)	0.039
Influenza season			
Cytomegalovirus	22 (11.3)	3 (3.4)	0.022
Influenza A virus	36 (18.5)	32 (36.8)	0.088
Influenza B virus	14 (7.2)	7 (8.0)	0.527
Rhinovirus	4 (2.1)	8 (9.2)	0.046
Respiratory syncytial virus	29 (14.9)	10 (11.5)	0.038
Adenovirus	3 (1.5)	0 (0)	0.157
Parainfluenza virus	4 (2.1)	3 (3.4)	0.857
Non-Influenza season			
Cytomegalovirus	67 (34.4)	1 (1.1)	<0.001
Influenza A virus	13 (6.7)	11 (12.6)	0.001
Influenza B virus	3 (1.5)	2 (2.3)	0.289
Rhinovirus	4 (2.1)	4 (4.6)	0.038
Respiratory syncytial virus	19 (9.7)	3 (3.4)	0.350
Adenovirus	4 (2.1)	5 (5.7)	0.009
Parainfluenza virus	10 (5.1)	2 (2.3)	0.696
Pathogenic types of coinfections	82 (42.1)	11 (12.6)	<0.001
Bacteria	27 (13.8)	3 (3.4)	0.009
* Streptococcus pneumoniae *	1 (0.5)	2 (2.3)	0.177
* Staphylococcus aureus *	7 (3.6)	0 (0)	0.074
* Escherichia coli *	3 (1.5)	0 (0)	0.245
* Enterobacter cloacae *	1 (0.5)	0 (0)	0.503
* Klebsiella pneumoniae *	6 (3.1)	1 (1.1)	0.337
* Pseudomonas *	6 (3.1)	0 (0)	0.098
* Proteus mirabilis *	1 (0.5)	0 (0)	0.503
* Acinetobacter *	1 (0.5)	0 (0)	0.503
* Nocardia *	1 (0.5)	0 (0)	0.503
*Atypical*	5 (2.6)	2 (2.3)	0.895
* Mycoplasma pneumoniae *	3 (1.5)	2 (2.3)	0.655
* Legionella *	2 (1.0)	0 (0)	0.404
Pneumocystis	25 (12.8)	0 (0)	<0.001
Aspergillus	23 (11.8)	6 (6.9)	0.211
Mycobacterium tuberculosis	1 (0.5)	0 (0)	0.503
Non-tuberculosis mycobacteria	1 (0.5)	0 (0)	0.503
Drug-resistant bacteria*	5/14	0/1	0.464
Pathogens types of nosocomial infection	69 (35.4)	9 (10.3)	<0.001
Bacteria	61 (31.3)	9 (10.3)	<0.001
* Acinetobacter *	19 (9.7)	3 (3.4)	0.069
* Pseudomonas *	10 (5.1)	3 (3.4)	0.534
* Klebsiella pneumoniae *	9 (4.6)	1 (1.1)	0.146
* Burkholderia *	4 (2.1)	0 (0)	0.178
* Enterococcus *	4 (2.1)	0 (0)	0.178
* Enterobacter cloacae *	2 (1.0)	0 (0)	0.343
* Escherichia coli *	2 (1.0)	0 (0)	0.343
* Enterobacter aerogenes *	0 (0)	1 (1.1)	0.134
* Stenotrophomonas maltophilia *	2 (1.0)	0 (0)	0.343
* Corynebacterium striatum *	3 (1.5)	0 (0)	0.245
* Staphylococcus aureus *	2 (1.0)	0 (0)	0.343
*Rolstonia mannitolytica*	0 (0)	1 (1.1)	0.134
*Other bacteria*	4 (2.1)	0 (0)	0.178
Aspergillus	8 (4.1)	0 (0)	0.055
Drug-resistant bacteria*	19/22	4/5	0.718

*Not all bacterial strains had drug-sensitivity results.

Patients with PIV had the highest average age (75 years) and the lowest incidence of fever (30.8 %). Patients with CMV and two or more virus groups had higher neutrophil and lactate dehydrogenase levels and lower lymphocyte counts than other viruses. Patients with CMV had a lower oxygenation index (*P*<0.05). Patients with CMV, HRV, PIV or two or more virus groups had more frequently required non-invasive mechanical ventilation, invasive mechanical ventilation and ICU care, and had higher rates of respiratory failure, septic shock and 30 day mortality (Table 3).

**Table 3.** Comparative analysis of different viral pneumonia in patients with interstitial lung disease

Variables	CMV *N*=64	IFV-A *N*=75	RSV *N*=47	IFV-B *N*=21	HPIV *N*=11	ADV *N*=10	HRV *N*=13	≥Two viruses *N*=41	*P*-Value
Female, *n* (%)	26 (40.6)	22 (29.3)	19 (40.4)	9 (42.9)	3 (27.3)	2 (20.0)	6 (46.2)	18(43.9)	0.567
Age, median (IQR), years	62.0 (49.5, 69.0)	68.0 (60.0, 74.0)	61.0 (53.0, 67.0)	66.0 (61.5, 71.5)	75.0 (68.0, 81.0)	64.0 (36.0, 70.8)	69.0 (64.0, 76.5)	65.0 (55.5, 69.5)	0.001
Symptoms and signs, *n* (%)									
Fever	53 (82.8)	47 (62.7)	25 (53.2)	14 (66.7)	6 (54.5)	6 (60.0)	4 (30.8)	26 (63.4)	0.008
Cough	57 (89.1)	74 (98.7)	47 (100.0)	18 (85.7)	11 (100.0)	10 (100.0)	13 (100.0)	40 (97.6)	0.013
Expectoration	54 (84.4)	71 (94.7)	46 (97.9)	16 (76.2)	11 (100.0)	10 (100.0)	11 (84.6)	37 (90.2)	0.031
Dyspnoea	53 (82.8)	53 (70.7)	37 (78.7)	13 (61.9)	9 (81.8)	10 (100.0)	9 (69.2)	34 (82.9)	0.179
Underlying diseases, *n* (%)									
Connective tissue disease	37 (57.8)	16 (21.3)	14 (29.8)	5 (23.8)	4 (36.4)	1 (10.0)	8 (61.5)	19 (46.3)	<0.001
Idiopathic interstitial pneumonia	21 (32.8)	40 (53.3)	17 (36.2)	14 (66.7)	4 (36.4)	4 (40.0)	10 (76.9)	11 (26.8)	0.002
Radiotherapy and chemotherapy of malignant solid	2 (3.1)	1 (1.3)	0 (0)	1 (4.8)	0 (0)	0 (0)	0 (0)	0 (0)	0.688
Solid organ transplant	0 (0)	8 (10.7)	12 (25.5)	0 (0)	2 (18.2)	1 (10.0)	0 (0)	7 (17.1)	0.001
Laboratory examination									
White blood cell,×10^9^ l^−1^ (IQR)	9.10 (6.04, 13.65)	7.69 (5.36, 10.77)	8.20 (5.88, 11.13)	5.83 (4.76, 7.91)	7.94 (4.70, 11.2)	7.19 (4.88, 12.27)	7.66 (6.17, 10.15)	8.30 (6.55, 11.15)	0.079
Neutrophils,×10^9^ l^−1^ (IQR)	7.12 (5.29, 11.50)	5.92 (3.73, 8.23)	5.70 (3.41, 8.91)	3.96 (2.77, 5.78)	5.53 (3.03, 7.68)	5.10 (2.83, 8.81)	5.88 (4.83, 6.93)	6.48 (4.97, 9.24)	0.004
Lymphocyte,×10^9^ l^−1^ (IQR)	0.90 (0.60, 1.40)	1.28 (0.72, 1.70)	1.16 (0.82, 2.11)	1.45 (0.90, 1.63)	1.00 (0.79, 1.37)	1.04 (0.86, 1.42)	1.44 (1.15, 2.03)	0.77 (0.33, 1.32)	0.002
Persistent lymphocytopenia	33 (51.6)	23(30.7)	13(27.7)	4(19.0)	3(27.3)	3(30.0)	4(30.8)	18 (43.9)	0.061
d-dimer, mg l^−1^	1.73 (0.73, 3.19)	0.55 (0.29, 1.82)	1.07 (0.52, 2.34)	0.40 (0.14, 0.79)	1.67 (0.80, 8.49)	0.87 (0.24, 1.53)	0.11 (0.03,0.17)	1.10 (0.60, 1.84)	<0.001
Lactate dehydrogenase, U l^−1^	373.0 (265.8, 516.6)	268.1 (210.6, 378.8)	301.0 (184.5, 398.5)	254.4 (202.0, 327.2)	247.0 (168.0, 336.0)	281.0 (234.0, 350.0)	250.1 (207.5, 378.7)	368.0 (274.0, 499.0)	0.003
Oxygenation index	204.3 (102.4, 282.1)	281.0 (210.0, 358.0)	314.3 (222.4, 423.8)	323.8 (272.0, 376.2)	306.7 (246.8, 421.9)	281.0 (125.6, 364.6)	220.5 (184.8, 327.5)	249.7 (128.7, 341.6)	<0.001
Severe pneumonia index score	87.0 (70.0, 121.3)	77.0 (64.0, 92.0)	68.0 (57.0, 82.0)	68.0 (54.5, 85.0)	88.0 (74.0, 102.0)	69.0 (34.8, 93.5)	68.0 (59.5, 71.5)	85.0 (64.5, 108.5)	<0.001
CURB65 score >1	24 (37.5)	19 (25.3)	6 (12.8)	5 (23.8)	3 (27.3)	3 (30.0)	1 (7.7)	14 (34.1)	0.092
Imaging features, *n* (%), 6 missing	1 (1.6)	1 (1.3)	0 (0)	0 (0)	0 (0)	0 (0)	1 (7.7)	3 (7.3)	
Consolidation	31 (49.2)	17 (23.0)	11 (23.4)	3 (14.3)	5 (45.5)	2 (20.0)	0 (0)	17 (41.5)	<0.001
Ground-glass opacity	49 (77.8)	42 (56.8)	33 (70.2)	12 (57.1)	5 (45.5)	5 (50.0)	10 (76.9)	25 (61.0)	0.084
Honeycomb or reticular pattern	44 (69.8)	56 (75.7)	35 (74.5)	6 (28.6)	10 (90.9)	4 (40.0)	8 (61.5)	27 (65.9)	0.002
Pleural effusion	12 (19.0)	9 (12.2)	6 (12.8)	0 (0)	1 (9.1)	2 (20.0)	0 (0)	4 (9.8)	0.358
Viral-PCP co-infection	21 (32.8)	0 (0)	0 (0)	1 (4.8)	0 (0)	0 (0)	0 (0)	3(7.3)	<0.001
Viral-aspergillus co-infection	6 (9.4)	9 (12.0)	4 (8.5)	0 (0)	3 (27.3)	0 (0)	`1 (7.7)	6 (14.6)	0.300
Viral-bacteria co-infection	10 (15.6）	5 (6.7)	5 (10.6)	0 (0	0 (0)	1 (10.0)	1 (7.7)	5 (12.2)	0.401
Viral-atypical co-infection	2 (3.1)	2 (2.7)	0 (0)	0 (0)	0 (0)	3 (30.0)	0 (0)	0 (0)	<0.001
Nosocomial bacterial infection	9 (14.1)	13 (17.3)	9 (19.1)	1 (4.8)	1 (9.1)	4 (40.0)	0 (0)	12 (29.3)	0.055
Complications, *n* (%)									
Non-invasive ventilation	31 (48.4)	9 (12.0)	7 (14.9)	3 (14.3)	3 (27.3)	1 (10.0)	3 (23.1)	10 (24.4)	<0.001
Invasive mechanical ventilation	22 (34.4)	16 (21.3)	10 (21.3)	1 (4.8)	3 (27.3)	1 (10.0)	2 (15.4)	15 (36.6)	0.064
Respiratory failure	49 (76.6)	33 (44.0)	16 (34.0)	3 (14.3)	3 (27.3)	4 (40.0)	5 (38.5)	24 (58.5)	<0.001
ICU care	42 (65.6)	17 (22.7)	11 (23.4)	1 (4.8)	3 (27.3)	1 (10.0)	3 (23.1)	17 (41.5)	<0.001
Septic shock during hospitalization	15 (23.4)	13 (17.3)	4 (8.5)	1 (4.8)	3 (27.3)	0 (0)	0 (0)	11 (26.8)	0.035
Extracorporeal membrane oxygenation	1 (1.6)	6 (8.0)	6 (12.8)	0 (0)	1 (9.1)	0 (0)	0 (0)	5 (12.2)	0.145
30 day mortality*	22 (34.4)	7 (9.3)	7 (14.9)	2 (9.5)	3 (27.3)	0 (0)	4 (30.8)	13 (31.7)	0.002

*The 30 day mortality between IFV and non-IFV patients was statistically different (9.4% vs 26.3%,*P*=0.001;13.5% vs 29.6%, *P*=0.003).

IFV, influenza A virus, influenza B virus; Non-IFV virus: respiratory syncytial virus (RSV), human parainfluenza virus (HPIV), human rhinovirus (HRV), adenovirus (AdV), and herpes simplex virus type 1(HSV-1); HSCT: hematopoietic stem cell transplantation. COPD: Chronic obstructive pulmonary disease.

The following parameters were significantly higher in the non-survivors' group than in the survivors' group: age, underlying connective tissue disease, proportion of fever and dyspnoea, peripheral blood leukocytes, neutrophils, lactate dehydrogenase, urea nitrogen, d-dimer on the first day of admission, patients with persistent lymphocytopenia, consolidation on CT image, PSI score and CURB-65 score >1,CMV infection, PCP infection, non-IFV infection, nosocomial bacterial infection, requirement for ICU admission, non-invasive ventilation, invasive mechanical ventilation and/or extracorporeal membrane oxygenation; respiratory failure; (*P*<0.05). Lymphocytes, haemoglobin, and albumin were significantly lower in the non-survivors' group than in the survivors' group (Table 4).

**Table 4.** Baseline characteristics of survivors and non-survivors 30 days after admission

Variables	Survivors, *n*=224	Non-survivors, *n*=58	*P*-value
Sex, female, *n* (%)	89 (39.7)	16 (27.6)	0.088
Age >60 years, *n* (%)	138 (61.6)	45 (77.6)	0.023
Symptoms and signs, *n* (%)			
Fever	134 (59.8)	47 (81.0)	0.003
Cough	213 (95.1)	57 (98.3)	0.284
Expectoration	204 (91.1)	52 (89.7)	0.740
Dyspnoea	165 (73.7)	53 (91.4)	0.004
Laboratory examination			
White blood cell,×10^9^ l^−1^ (IQR)	7.54 (5.61–10.63)	9.27 (6.78–12.27)	0.004
Neutrophils,×10^9^ l^−1^ (IQR)	5.70 (3.51–8.25)	7.05 (5.66–10.41)	<0.001
Lymphocyte,×10^9^ l^−1^ (IQR)	1.16 (0.73–1.70)	0.79 (0.59–1.23)	0.003
Persistent lymphocytopenia	66 (29.5)	35 (60.3)	<0.001
Mean hemoglobin ±sd, g l^−1^	122.3±23.0	113.5±25.5	0.012
Mean albumin ±sd, g l^−1^	35.9±5.0	32.1±5.7	<0.001
Lactate dehydrogenase, U l^−1^	293 (213–397)	373 (256–502)	0.009
Blood urea nitrogen, mmol l^−1^	5.25 (4.10–7.69)	6.55 (5.26–11.14)	0.008
d-dimer, mmol l^−1^	0.78 (0.32–1.84)	1.35 (0.44–4.99)	0.014
Procalcitonin, ng ml^−1^	0.24 (0.09–0.38)	0.24 (0.10–0.47)	0.730
Oxygenation index	288.9 (211.6–375.9)	145.0 (106.3–247.7)	<0.001
Severe pneumonia index score	72 (62–90)	91 (73–126)	<0.001
CURB65 score >1	51 (22.8)	24 (41.4)	0.004
Underlying diseases, *n* (%)			
Diabetes mellitus	62 (27.7)	18 (31.0)	0.613
Connective tissue disease*	76 (33.9)	28 (48.3)	0.044
Idiopathic pulmonary fibrosis	95 (42.4)	26 (44.8)	0.740
Chronic obstructive pulmonary disease	23 (10.3)	2 (3.4)	0.103
Radiotherapy and chemotherapy of malignant solid tumour	2 (0.9)	2 (3.4)	0.142
Unilateral lung transplantation^&^	27 (12.1)	3 (5.2)	0.130
Current smoker or ex-smoker	86 (38.4)	23 (39.7)	0.860
Bronchoalveolar lavage, *n* (%)	130 (58.0)	27 (46.6)	0.117
Imaging features, *n* (%), 6 missing	220 (98.2)	56 (96.6)	-
Consolidation	59 (26.8)	27 (48.2)	0.002
Ground-glass opacity	142 (64.5)	39 (69.4)	0.473
Honeycomb or Reticular pattern	146 (66.3)	44 (78.6)	0.085
Pleural effusion	26 (11.8)	8 (14.3)	0.591
Two or more viruses	13 (5.8)	28 (48.3)	0.056
Cytomegalovirus	63 (28.1)	30 (51.7)	0.001
Non-influenza virus	124 (55.4)	43 (74.1)	0.009
Viral-PCP co-infection	16 (7.1)	9 (15.5)	0.046
Viral-aspergillus co-infection	21 (9.4)	8 (13.8)	0.324
Viral-bacteria co-infection	23 (10.3)	4 (6.9)	0.437
Viral-atypical co-infection	6 (2.7)	1 (1.7)	0.677
Nosocomial bacterial infection	33 (14.7)	16 (27.6)	0.021
Complications, *n* (%)			
Non-invasive ventilation	33 (14.7)	34 (58.6)	<0.001
Invasive mechanical ventilation	34 (15.2)	36 (62.1)	<0.001
Mechanical ventilation	57 (25.4)	42 (72.4)	<0.001
Respiratory failure	84 (37.5)	53 (91.4)	<0.001
ICU admission	52 (23.2)	43 (74.1)	<0.001
Extracorporeal membrane oxygenation	13 (5.8)	6 (10.3)	0.219

**Fig. 1.**
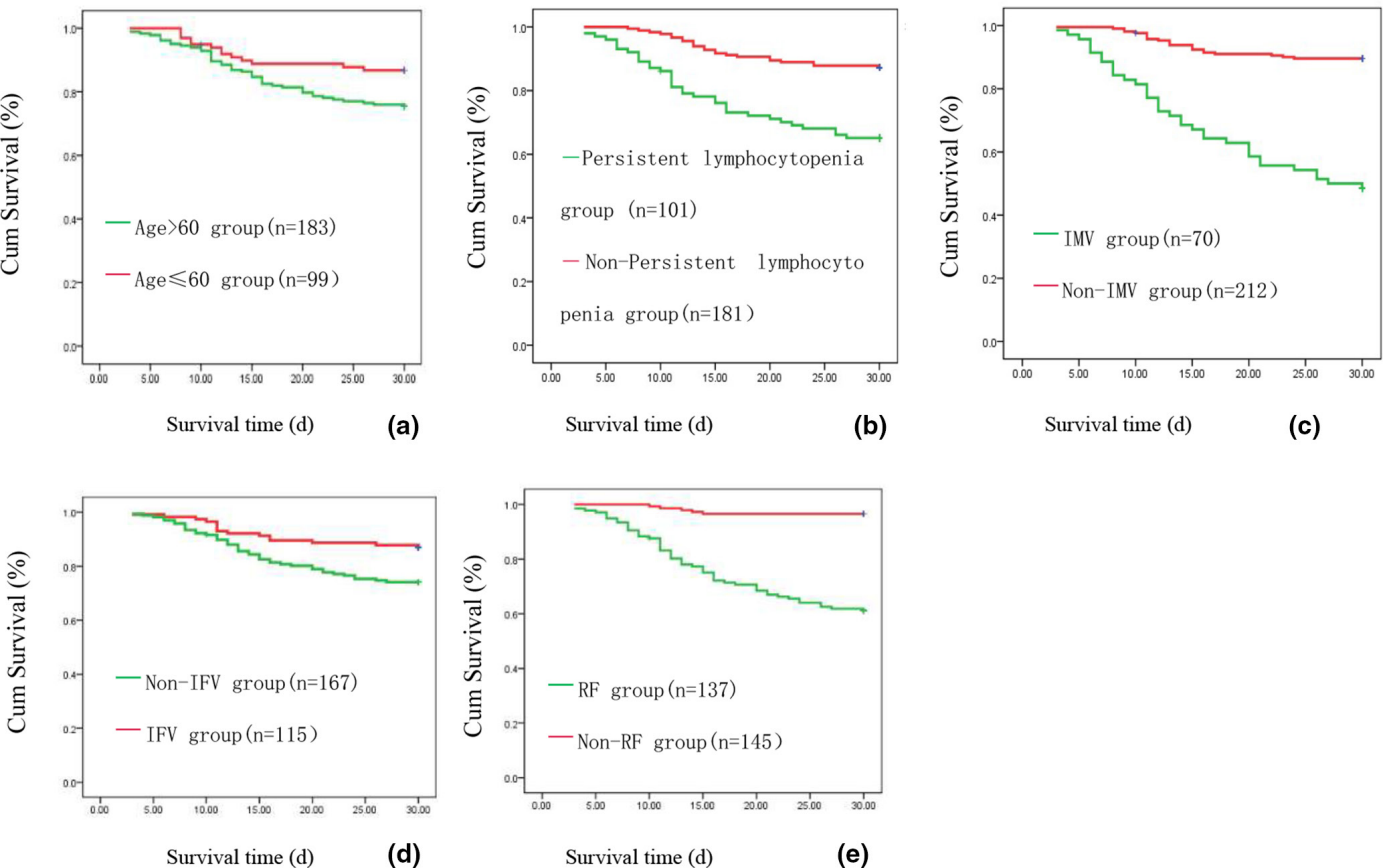
Survival curve of patients with viral pneumonia in interstitial lung disease. Survival curve of patients with viral pneumonia in interstitial lung disease. (a) The 30 day survival curve of age >60 years and age ≤60 years group; (b) The 30 day survival curve of persistent lymphocytopenia and non-persistent lymphocytopenia group; (c) The 30 day survival curve of invasive mechanical ventilation (IMV) group and non-IMV group; (d) The 30 day survival curve of influenza A virus (FluA) group and non-FluA group; (e) The 30 day survival curve of respiratory failure (RF) group and non-RF group (all *P*<0.05).

Multivariate Cox regression analysis indicated that the following factors were independent predictors of 30 day mortality in patients with ILD: age >60 years, respiratory failure, persistent lymphocytopenia, invasive mechanical ventilation and non-IFV type A infection (Table 5, Fig. 1).

**Table 5.** Variables associated with 30 day mortality in patients with interstitial lung disease

*Variables*	Univariate analysis			Multivariate analysis		
	HR	95 % CI	*P*-value	HR	95 % CI	*P*-value
Age >60	1.961	1.058–3.634	0.033	2.722	1.286–5.762	0.009
Sex	1.607	0.903–2.858	0.107			
Fever	2.639	1.368–5.088	0.004			
Consolidation on CT image	2.256	1.335–3.810	0.002			
Persistent lymphocytopenia	3.214	1.898–5.441	<0.001	2.017	1.083–3.757	0.027
Invasive mechanical ventilation	6.377	3.747–10.853	<0.001	3.328	1.645–6.734	0.001
Non-influenza virus	2.559	1.295–5.509	0.007	3.184	1.557–6.512	0.002
Nosocomial bacterial infection	1.928	1.084–3.429	0.026			
Connective tissue disease	1.667	0.996–2.790	0.052			
Pneumocystis	2.330	1.144–4.746	0.020			
Cytomegalovirus	2.413	1.441–4.040	0.001			
Two or more viruses	1.852	0.999–3.433	0.050			
CURB65 >1	2.114	1.254–3.566	0.005			
PSI	1.021	1.012–1.029	<0.001			
Intensive care unit admission	7.029	3.901–12.664	<0.001			
Leukocytes on the first day of admission	1.063	1.015–1.114	0.010			
Neutrophils on the first day of admission	1.085	1.033–1.139	0.001			
Lymphocytes on the first day of admission	0.544	0.355–0.832	0.005			
Haemoglobin on the first day of admission	0.988	0.979–0.998	0.017			
Albumin on the first day of admission	0.886	0.843–0.931	<0.001			
Lactate dehydrogenase	1.001	1.000–1.002	0.017			
d-dimer on the first day of admission	1.084	1.030–1.140	0.002			
Respiratory failure	13.790	5.509–34.518	<0.001	5.165	1.838–14.515	0.002

## Discussion

This study was a large-scale retrospective investigation of the clinical characteristics and prognostic risk factors of mortality in hospitalized patients with ILD who developed viral infection. The main findings are summarized as follows: (1) patients with ILD who developed viral infection had a higher mortality, with the 30 day rates being 20.6 %, respectively; (2) the distribution of virus types in immunocompromised patients differed between influenza and non-influenza seasons; (3) the disease severity and mortality in non-IFV patients were higher than those of IFV patients; and (4) independent risk factors for mortality included age >60 years, respiratory failure, persistent lymphocytopenia, invasive mechanical ventilation and non-IFV infection.

Previous studies have shown that viruses, especially respiratory viruses, may be co-factors for the development or exacerbation of lung fibrosis [15]. One such study, which conducted autopsies in 42 patients with IPF, reported that 15 % had a fungal, bacterial and/or viral infection [16]. Another study found that 28.8 % of patients with an acute exacerbation of IPF, had bronchopneumonia (fungal, 13.5 %; CMV, 11.5 %; and bacterial, 9.6 %) [17]. Wootton *et al.* reported that only 4 of 43 patients with an acute exacerbation of IPF had evidence of common respiratory viral infections (PIV [*n*=1], HRV [*n*=2], coronavirus [*n*=1]) [18]. Similarly, in a study conducted among 40 patients with IPF, Keyvani *et al.* documented infections in nine patients (22.5%); RSV, PIV, HRV and coronaviruses were found in 2.5 %(1/40), 7.5 % (3/40), 10 %(4/40) and 2.5 %(1/40) of the patients, respectively [19]. Our large-scale epidemiological study of patients with ILD and viral infection found that IFV and RSV were the main pathogens during the influenza season, followed by CMV. During the non-influenza season, CMV was the main pathogen in immunocompromised patients, followed by IFV, RSV, PIV and HRV. Therefore, in the case of patients with suspected interstitial disease complicated with virus infection, we suggest that the viral nucleic acid test should be performed as early as possible to confirm the etiological diagnosis.

The disease severity, complications, and outcomes of immunocompetent patients with community-acquired pneumonia were similar between IFV and non-IFV-related respiratory diseases [20–22]. For elderly hospitalized patients with respiratory symptoms, RSV, human metapneumovirus and PIV have been associated with higher mortality [23–26] and more complications [25] than influenza. Our study showed that disease severity and mortality in non-IFV patients were higher than those in IFV patients. This result can be attributed to the following reasons: (1) the early use of oseltamivir in patients with influenza; (2) the lack of a specific drug for HRV and PIV; and (3) CMV was closely related to immunocompromised patients and had high mortality [27, 28]. Thus, when patients with ILD develop symptoms of a viral infection, an increased vigilance is warranted for the detection of non-IFV infections.

Factors identified by previous studies as being associated with a poor prognosis in patients with ILD include a lower baseline forced vital capacity and carbon monoxide diffusing capacity; more extensive abnormalities on computed tomography at the time of acute exacerbation; and poor oxygenation and BALF neutrophil and lymphocyte percentages [29, 30]. Viral infections, mostly CMV and human herpesvirus 7, have been identified in patients with acute exacerbation of IPF and non-IPF ILDs; however, virus infection was not found to be an independent predictor of 60 day survival in a simple logistic regression analysis [5]. Moua *et al.* suggested that the following factors were predictive of increased in-hospital mortality: male sex, acute exacerbation, longer duration of hospitalization, ICU admission, mechanical ventilation, use of bronchoscopy in an ICU setting and the intravenous administration of high-dose steroids [1]. In our study, we did not find a close relationship between high-dose hormone administration and poor prognosis, but we found that lymphopenia was directly related to poor prognosis, similar to the finding of other viral infection studies [31]. We also found that non-IFV virus infection was closely related to poor prognosis. Therefore, we must pay attention to the higher mortality rates due to viral infections such as CMV, HRV, PIV and mixed virus infections.

This study had several limitations. First, it utilized a retrospective observational design. Second, lung-function tests were not performed, as many of the patients could not undergo these tests. Third, we did not re-evaluate patient prognosis at a 1 year follow-up; therefore, it was impossible to suggest that viral infection was associated with poor long-term prognosis of ILD.

## Conclusions

Patients with ILD who subsequently developed viral infection had high rates of morbidity and mortality, which were associated with increased age (>60 years), respiratory failure, mechanical ventilation, persistent lymphocytopenia and non-IFV virus infection. These risk factors should be carefully considered when determining treatment strategies for this patient population.
